# Potential causal association between mucosal proctocolitis and Alzheimer’s disease: A Mendelian Randomization Study

**DOI:** 10.1002/alz.094721

**Published:** 2025-01-09

**Authors:** Dinghao An, Yun Xu

**Affiliations:** ^1^ Peking Union Medical College, Beijing China; ^2^ Affiliated Drum Tower Hospital of Medical School, Nanjing University, NANJING, Jiangsu China

## Abstract

**Background:**

The gut microbiota plays a certain role in Alzheimer’s disease, and various types of enteritis can also cause disturbances in the gut microbiota. Recently, researchers have substantially highlighted the effect of some enteritis on Alzheimer’s disease incidence and development. In this study, we elucidated the correlation between mucosal proctocolitis and Alzheimer’s disease and possible mechanism.

**Method:**

The association between mucosal proctocolitis and the risk of Alzheimer’s disease was evaluated by using two‐sample Mendelian randomization(MR), Five methods were used to perform MR analysis: the inverse variance‐weighted, weighted median, MR‐Egger, Simple mode, and Weighted mode methods. Furthermore, Heterogeneity test, MR‐Egger intercept test, MR Pleiotropy Residual Sum and Outlier test, and leave‐one‐out analysis were performed to analyze sensitivity. MR‐PRESSO and MR‐RAPS were performed to further evaluate the robustness.

**Result:**

After implementing multiple test corrections, we observed that genetic susceptibility to mucosal proctocolitis was associated with an increased risk of Alzheimer’s disease. However, a causal relationship between mucosal proctocolitis and markers of Alzheimer’s disease was not observed. We also found mucosal proctocolitis can also affect a certain microbial community that can cause a high risk of developing Alzheimer’s disease. Sensitivity analysis confirmed the robustness of the results.

**Conclusion:**

We confirmed the causal relationship between mucosal proctocolitis and Alzheimer’s disease, thereby providing a novel viewpoint on the prevention and treatment of Alzheimer’s disease. However, we did not observe any AD‐related gut microbiota may play a mediating role. Our findings suggest the integration of intestinal system protection and maintenance of intestinal probiotics health into routine clinical care to enhance the effectiveness of Alzheimer’s disease treatment.

